# Supplementary material for: Association between land use, land cover, plant genera, and pollinator abundance in mixed-use landscapes
Source: PLoS One. 2023 Nov 22;18(11):e0294749. doi: 10.1371/journal.pone.0294749 (PMC10664889; doi:10.1371/journal.pone.0294749)
Supplement: S1 File — (DOCX) [file pone.0294749.s001.docx]

**Supplementary Information**

**Table S1.** List of birds (Honeyeaters) observed across the different land use.

| Land use | Plot Code | Coordinates | | **Birds (Honeyeaters)** | | | | | | | |
| --- | --- | --- | --- | --- | --- | --- | --- | --- | --- | --- | --- |
|  |  | X | Y | *Melithreptus*  *affinis* | *Melithreptus validirostris* | *Nesoptilotis flavicollis* | *Anthochaera paradoxa* | *Anthochaera chrysoptera* | *Phylidonyris novaehollandiae* | *Phylidonyris pyrrhopterus* | *Acanthorhynchus tenuirostris* |
| Protected area | LX1 | 147.694525 | -42.95973798 |  |  | 4 | 3 | 8 | 17 | 2 |  |
|  | LX2 | 147.689147 | -42.95929802 | 5 |  | 5 | 10 | 3 | 2 | 3 |  |
|  | LX3 | 147.685683 | -42.96248297 |  |  | 3 |  | 3 |  | 1 |  |
|  | LY1 | 147.714192 | -42.95687699 |  |  | 8 | 5 | 3 | 8 | 1 |  |
|  | LY2 | 147.720296 | -42.95969901 | 1 |  | 6 | 1 |  |  |  |  |
|  | LY4 | 147.722514 | -42.96706603 |  |  | 5 |  | 1 | 3 | 4 |  |
|  | FX0 | 147.957763 | -43.13124698 | 2 | 1 | 4 | 4 | 1 | 1 | 2 | 2 |
|  | FX2 | 147.960053 | -43.12377602 | 2 |  | 1 | 2 | 4 |  | 7 |  |
|  | FX4 | 147.967787 | -43.12231899 |  |  | 2 | 3 |  |  | 2 |  |
|  | FX7 | 147.973843 | -43.11870597 |  |  | 4 |  |  |  | 3 |  |
|  | FX8 | 147.954377 | -43.13355996 | 1 |  | 2 | 2 |  |  | 5 |  |
|  | FX9 | 147.954482 | -43.13768503 | 2 | 5 | 1 | 1 |  |  | 1 |  |
|  | TX1 | 147.736919 | -43.13311203 |  |  | 1 |  |  |  |  |  |
|  | TX2 | 147.742397 | -43.13633303 |  |  |  |  |  |  | 5 |  |
|  | TX3 | 147.747442 | -43.13836204 |  | 7 |  |  |  |  | 7 |  |
|  | TY0 | 147.719269 | -43.148077 |  |  | 4 |  |  |  |  |  |
|  | TY1 | 147.717747 | -43.153528 |  |  | 2 | 2 | 2 |  | 2 |  |
|  | TY2 | 147.723688 | -43.15582397 |  |  |  | 1 |  |  | 3 |  |
|  |  |  |  |  |  |  |  |  |  |  |  |
| Plantation | EX2 | 147.808236 | -43.16630504 |  |  | 1 | 5 |  |  | 7 |  |
|  | EX3 | 147.812878 | -43.17359998 |  |  |  |  |  |  | 5 |  |
|  | EX4 | 147.816944 | -43.17799302 |  | 6 |  |  |  |  | 6 | 1 |
|  | EY1 | 147.792215 | -43.17746697 |  |  | 1 |  |  |  | 7 |  |
|  | EY2 | 147.789374 | -43.18006896 |  |  |  |  |  |  | 5 |  |
|  | EY3 | 147.797402 | -43.183167 |  |  | 2 |  |  |  | 15 |  |
|  | NX1 | 147.777547 | -43.164141 |  |  |  |  |  |  | 4 |  |
|  | NX2 | 147.774523 | -43.16773902 |  |  |  |  |  |  | 2 |  |
|  | NX3 | 147.770561 | -43.17086999 |  |  |  |  |  |  | 8 |  |
|  | NY1 | 147.765693 | -43.18480901 |  |  |  |  |  |  | 1 |  |
|  | NY2 | 147.761728 | -43.18323598 |  |  |  |  |  |  | 3 |  |
|  | NY3 | 147.757024 | -43.18636402 |  |  |  |  |  |  |  |  |
|  |  |  |  |  |  |  |  |  |  |  |  |
| Pasture | PX2 | 147.783353 | -43.05844101 |  |  |  |  |  |  |  |  |
|  | PX3 | 147.783353 | -43.06262299 |  |  |  |  |  |  |  |  |
|  | PX4 | 147.785901 | -43.06890699 |  |  |  |  |  |  |  |  |
|  | PY2 | 147.748583 | -43.04908304 |  |  |  |  |  |  |  |  |
|  | PY3 | 147.747694 | -43.05269396 |  |  |  |  |  |  |  |  |
|  | PY4 | 147.746351 | -43.05648501 |  |  |  |  |  |  |  |  |

**Table S2.** List of bees observed across the different land use.

| Land use | Plot Code | Coordinates | | **Bees** | | | | |
| --- | --- | --- | --- | --- | --- | --- | --- | --- |
|  |  |  |  | **Native bees** | | | **Introduced Bees** | |
|  |  | X | Y | *Exoneura* | *Lasioglossum* | Others | *Apis mellifera* | *Bombus terrestris* |
| Protected area | LX1 | 147.694525 | -42.95973798 | 10 | 2 | 6 | 1 |  |
|  | LX2 | 147.689147 | -42.95929802 | 15 | 2 | 2 | 1 |  |
|  | LX3 | 147.685683 | -42.96248297 | 6 | 3 | 4 | 5 |  |
|  | LY1 | 147.714192 | -42.95687699 | 20 | 2 |  | 14 |  |
|  | LY2 | 147.720296 | -42.95969901 |  |  | 5 | 1 |  |
|  | LY4 | 147.722514 | -42.96706603 | 3 |  |  |  | 1 |
|  | FX0 | 147.957763 | -43.13124698 |  |  |  | 12 | 2 |
|  | FX2 | 147.960053 | -43.12377602 | 4 |  | 1 | 3 |  |
|  | FX4 | 147.967787 | -43.12231899 | 3 |  |  |  |  |
|  | FX7 | 147.973843 | -43.11870597 |  |  |  |  |  |
|  | FX8 | 147.954377 | -43.13355996 | 3 |  |  | 3 |  |
|  | FX9 | 147.954482 | -43.13768503 | 1 |  |  | 4 |  |
|  | TX1 | 147.736919 | -43.13311203 | 7 | 1 | 2 | 14 |  |
|  | TX2 | 147.742397 | -43.13633303 | 12 | 2 | 5 | 9 | 2 |
|  | TX3 | 147.747442 | -43.13836204 | 10 | 2 |  | 8 | 1 |
|  | TY0 | 147.719269 | -43.148077 | 20 |  | 1 | 11 |  |
|  | TY1 | 147.717747 | -43.153528 | 3 | 1 | 6 | 10 | 1 |
|  | TY2 | 147.723688 | -43.15582397 | 3 |  |  | 13 | 2 |
|  |  |  |  |  |  |  |  |  |
| Plantation | EX2 | 147.808236 | -43.16630504 | 5 |  | 1 | 3 |  |
|  | EX3 | 147.812878 | -43.17359998 |  |  |  |  |  |
|  | EX4 | 147.816944 | -43.17799302 |  |  | 3 | 7 | 2 |
|  | EY1 | 147.792215 | -43.17746697 |  |  |  | 2 |  |
|  | EY2 | 147.789374 | -43.18006896 |  |  |  | 1 |  |
|  | EY3 | 147.797402 | -43.183167 |  |  |  |  |  |
|  | NX1 | 147.777547 | -43.164141 | 8 | 1 | 2 | 10 |  |
|  | NX2 | 147.774523 | -43.16773902 | 6 |  | 1 | 12 |  |
|  | NX3 | 147.770561 | -43.17086999 | 6 |  | 1 | 8 |  |
|  | NY1 | 147.765693 | -43.18480901 |  |  |  |  |  |
|  | NY2 | 147.761728 | -43.18323598 | 5 | 1 |  |  | 2 |
|  | NY3 | 147.757024 | -43.18636402 | 7 | 3 |  |  | 1 |
|  |  |  |  |  |  |  |  |  |
| Pasture | PX2 | 147.783353 | -43.05844101 |  | 5 |  | 4 | 1 |
|  | PX3 | 147.783353 | -43.06262299 |  | 9 |  | 4 |  |
|  | PX4 | 147.785901 | -43.06890699 | 27 | 3 | 10 | 26 |  |
|  | PY2 | 147.748583 | -43.04908304 |  | 6 | 2 | 14 | 1 |
|  | PY3 | 147.747694 | -43.05269396 |  | 2 |  | 10 |  |
|  | PY4 | 147.746351 | -43.05648501 |  | 3 |  | 1 |  |

**Table S3.** List of beetles observed across the different land use.

| Land use | Plot Code | Coordinates | | **Beetle** | | | | | | | | |
| --- | --- | --- | --- | --- | --- | --- | --- | --- | --- | --- | --- | --- |
|  |  | X | Y | *Tenebrionidae* | *Scarabaeidae* | *Buprestidae* | *Oedemeridae* | *Elateridae* | *Cantharidae* | *Lycidae* | *Cleridae* | *Cerambycidae* |
| Protected area | LX1 | 147.694525 | -42.95973798 |  | 1 | 1 | 5 |  |  | 1 |  | 1 |
|  | LX2 | 147.689147 | -42.95929802 |  | 9 |  | 7 |  |  | 1 |  | 1 |
|  | LX3 | 147.685683 | -42.96248297 |  | 3 |  | 17 |  |  | 11 |  |  |
|  | LY1 | 147.714192 | -42.95687699 |  | 3 |  | 10 |  |  |  | 2 |  |
|  | LY2 | 147.720296 | -42.95969901 |  | 22 |  | 7 |  |  |  |  | 3 |
|  | LY4 | 147.722514 | -42.96706603 |  | 3 |  |  |  |  |  |  |  |
|  | FX0 | 147.957763 | -43.13124698 |  |  |  |  |  |  |  |  |  |
|  | FX2 | 147.960053 | -43.12377602 |  |  |  |  |  |  |  |  |  |
|  | FX4 | 147.967787 | -43.12231899 |  |  |  | 3 |  |  |  |  |  |
|  | FX7 | 147.973843 | -43.11870597 |  |  |  |  |  |  |  |  |  |
|  | FX8 | 147.954377 | -43.13355996 |  |  |  | 2 |  |  |  |  |  |
|  | FX9 | 147.954482 | -43.13768503 |  |  |  | 1 |  |  |  |  |  |
|  | TX1 | 147.736919 | -43.13311203 |  | 46 |  | 24 |  |  | 8 | 10 | 1 |
|  | TX2 | 147.742397 | -43.13633303 |  |  | 1 | 2 |  |  |  |  |  |
|  | TX3 | 147.747442 | -43.13836204 | 1 |  |  | 3 | 1 |  |  |  |  |
|  | TY0 | 147.719269 | -43.148077 | 5 | 1 |  | 3 |  |  | 7 |  |  |
|  | TY1 | 147.717747 | -43.153528 |  |  |  | 18 |  |  | 1 | 1 |  |
|  | TY2 | 147.723688 | -43.15582397 |  | 1 | 3 | 3 |  |  | 1 |  |  |
|  |  |  |  |  |  |  |  |  |  |  |  |  |
| Plantation | EX2 | 147.808236 | -43.16630504 |  |  |  | 10 |  |  |  |  |  |
|  | EX3 | 147.812878 | -43.17359998 |  |  |  |  |  |  |  |  |  |
|  | EX4 | 147.816944 | -43.17799302 |  |  |  | 5 |  |  |  |  |  |
|  | EY1 | 147.792215 | -43.17746697 |  |  |  |  |  |  |  |  |  |
|  | EY2 | 147.789374 | -43.18006896 |  |  |  |  |  |  |  |  |  |
|  | EY3 | 147.797402 | -43.183167 |  |  |  | 11 |  |  |  |  |  |
|  | NX1 | 147.777547 | -43.164141 |  |  |  | 20 |  | 57 | 2 |  |  |
|  | NX2 | 147.774523 | -43.16773902 |  |  |  |  |  | 4 |  |  |  |
|  | NX3 | 147.770561 | -43.17086999 | 1 |  |  |  |  | 40 |  |  |  |
|  | NY1 | 147.765693 | -43.18480901 |  |  |  |  |  |  |  |  |  |
|  | NY2 | 147.761728 | -43.18323598 | 1 |  |  | 5 |  |  |  |  |  |
|  | NY3 | 147.757024 | -43.18636402 |  |  |  | 7 |  |  |  |  |  |
|  |  |  |  |  |  |  |  |  |  |  |  |  |
| Pasture | PX2 | 147.783353 | -43.05844101 |  |  |  |  |  |  |  |  |  |
|  | PX3 | 147.783353 | -43.06262299 |  |  |  |  |  |  |  |  |  |
|  | PX4 | 147.785901 | -43.06890699 | 5 |  |  |  |  |  |  |  |  |
|  | PY2 | 147.748583 | -43.04908304 |  |  |  |  |  |  |  |  |  |
|  | PY3 | 147.747694 | -43.05269396 |  |  |  |  |  |  |  |  |  |
|  | PY4 | 147.746351 | -43.05648501 |  |  |  |  |  |  |  |  |  |

**Table S4.** Confusion matrix of the classified land-cover image which includes forest, open, barren and water classes. Reference and prediction refer to the validation data and the predicted land-cover image, respectively.

|  | | Reference | | | |
| --- | --- | --- | --- | --- | --- |
|  |  | Forest | Open | Barren | Water |
| Prediction | Forest | 96 | 2 | 2 | 0 |
|  | Open | 6 | 87 | 7 | 0 |
|  | Barren | 1 | 19 | 79 | 1 |
|  | Water | 28 | 16 | 0 | 56 |

**Table S5.** Sensitivity and specificity of each of the land-cover classes.

|  | Land cover | | | |
| --- | --- | --- | --- | --- |
|  | Forest | Open | Barren | Water |
| Sensitivity | 0.7328 | 0.7016 | 0.8977 | 0.9825 |
| Specificity | 0.9851 | 0.9529 | 0.9327 | 0.8717 |

**Table S6.** Correlation of fixed effects in the honeyeater model.

|  | Intercept | LU_protected.area | LU_plantation | Eucalyptus | Symphyomyrtus | log(1+forest.100) |
| --- | --- | --- | --- | --- | --- | --- |
| LU_protected.area | 0.224 |  |  |  |  |  |
| LU_plantation | 0.264 | 0.929 |  |  |  |  |
| Eucalyptus | -0.168 | -0.331 | -0.401 |  |  |  |
| Symphyomyrtus | 0.022 | -0.352 | -0.128 | 0.178 |  |  |
| log(1+forest.100) | -0.65 | -0.757 | -0.777 | 0.014 | -0.024 |  |
| log(1+open.1500) | -0.784 | 0.14 | 0.097 | 0.227 | -0.012 | 0.145 |

**Table S7.** Correlation of fixed effects in the native bee model.

|  | Intercept | genera_Pultenaea | genera_Leucopogon |
| --- | --- | --- | --- |
| genera_Pultenaea | -0.128 |  |  |
| genera_Leucopogon | -0.103 | 0.104 |  |
| log(1+open.250) | -0.878 | 0.013 | 0.024 |

**Table S8.** Correlation of fixed effects in the introduced bee model.

|  | Intercept | LU_pasture | log  (1+open.250) | genera_Pomaderris | genera_Leptospermum | genera_Lissanthe | genera_Acacia | genera_Pimelea | genera_Melaleuca |
| --- | --- | --- | --- | --- | --- | --- | --- | --- | --- |
| LU_pasture | 0.507 |  |  |  |  |  |  |  |  |
| log(1+open.250) | -0.863 | -0.77 |  |  |  |  |  |  |  |
| genera_Pomaderris | -0.025 | 0.184 | -0.138 |  |  |  |  |  |  |
| genera_Leptospermum | -0.029 | 0.12 | -0.08 | 0.097 |  |  |  |  |  |
| genera_Lissanthe | -0.137 | 0.02 | 0.045 | 0.075 | 0.011 |  |  |  |  |
| genera_Acacia | -0.037 | 0.112 | -0.107 | 0.083 | 0.207 | 0.008 |  |  |  |
| genera_Pimelea | -0.08 | 0.203 | -0.122 | 0.186 | 0.191 | 0.055 | 0.245 |  |  |
| genera_Melaleuca | -0.036 | 0.177 | -0.127 | 0.182 | 0.1 | 0.054 | 0.203 | 0.172 |  |
| genera_Olearia | -0.322 | -0.101 | 0.226 | 0.055 | 0.044 | 0.068 | 0.053 | 0.101 | 0.05 |

**Table S9.** Correlation of fixed effects in the beetle model.

|  | Intercept |
| --- | --- |
| genera_Leptospermum | -0.217 |


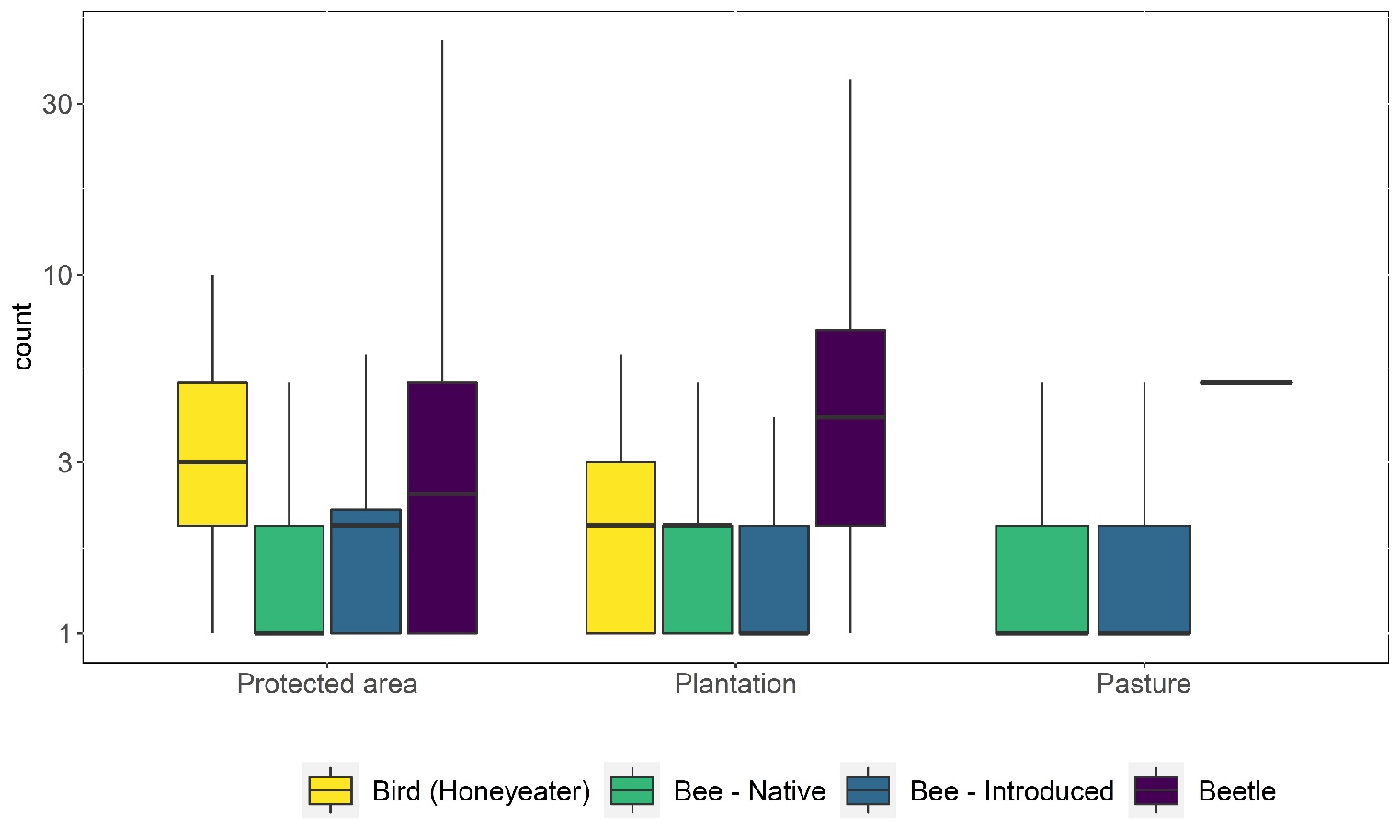


**Fig S1.** The median count (along a logarithmic axis) of birds (honeyeaters), bees (native and introduced), and beetles within the different land use – protected area, plantation, and pasture.


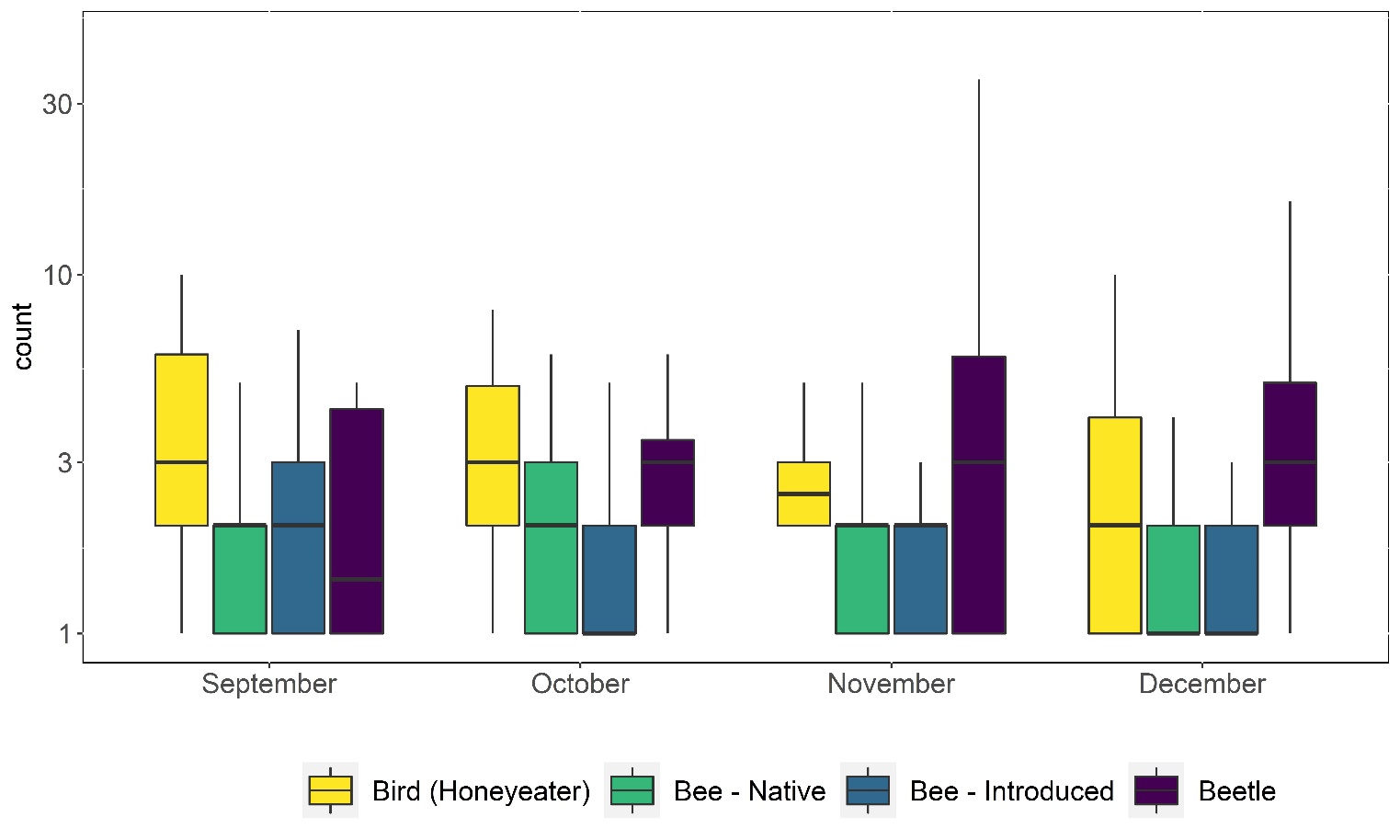


**Fig S2.** The median count (along a logarithmic axis) of birds (honeyeaters), bees (native and introduced), and beetles across the different months – September, October, November, and December.
