# Supplementary material for: Association between land use, land cover, plant genera, and pollinator abundance in mixed-use landscapes
Source: PLoS One. 2023 Nov 22;18(11):e0294749. doi: 10.1371/journal.pone.0294749 (PMC10664889; doi:10.1371/journal.pone.0294749)
Supplement: Attachment — Submitted filename: PONE Rebuttal 2.2.docx [file pone.0294749.s002.docx]

**Rebuttal**

Association between land use, land cover, plant genera, and pollinator abundance in mixed-use landscapes

Authors: Vishesh L. Diengdoh, Barry W. Brook, Mark Hunt, Stefania Ondei

Note: The reviewer’s text is in blue while the authors text is in black for clarity. New text by the author are in italics.

Reviewer #2: The manuscript has been improved substantially. However, given the modified results sections, the discussion needs to follow these changes sufficiently. I suggest modifying the discussion to explain the results adequately.

The discussion section does not sufficiently discuss the result of the effect of plant genera identity on pollinator abundance. It would be important to add predictions or explanations on what attributes of the plant genera might be crucial in explaining the obtained results.

1. We have now discussed the influence of plant genera on the obtained results.

*L348:* *Although the partial R^2^ for most plant genera were zero in the insect models, some had positive albeit very small R^2^ values, suggesting a potential weak relationship between insect abundance and some plant genera. These relationships have plausible biological underpinnings. Pultenaea and Pomaderris are pollinated by bees, beetles, and butterflies (77-79). Further, the zygomorphic shape of Pultenaea flowers could be important for native bees, as this taxon has been shown to exhibit bee-pollination syndrome (i.e. flower features evolved in response to natural selection driven by bees), although pollinator syndromes have been found to be an inadequate explanation for pollinator visitation in Tasmania (80). Pimelea spp. have narrow tubular odourless flowers which are known to be visited by introduced bees – Apis mellifera and Bombus terrestris – which have long tongues (80). Leptospermum, in addition to providing nectar and pollen, is also a rich source of fruits and grass-root material which beetles and their larvae feed upon (81, 82). The actinomorphic shape of Leptospermum could facilitate beetle visitation, as they find it easier to land on simple dish- and bowl-shaped blossoms (79).*

Table 1 and analysis: Were the interactive effects of different predictors tested?

2. No, the interactive effects of the different predictors were not tested. We included all predictors that had a non-zero R^2^ value.

If we assessed and compared the interactive effects of models having an unequal number of predictors, models with a greater number of predictors would have equal or higher R^2^ values but be overfitted.

Line 310: Given that the models or their strength is very low in drawing conclusions apart from honeyeaters, what is the confidence level’ that one can place on results obtained from the other species i.e. bees and beetles?

3. Since the R^2^ for the bee and beetle models are low, we carefully avoided making any bold conclusions regarding the results for these taxa, and their limitations have been covered.

For example, in L312-324 we focused on land-types and honeyeaters only, while L324-331 focused on the field observations of the insects and not the models. We provided potential explanations for the low R^2^ values in L361-367.

Although in L385 we made recommendations of plant species management for promoting and sustaining the abundance of bees and beetles, we have now re-worded them to better reflect our results.

*L386:* *Based on our results for the Tasman Peninsula, plants species belonging to the native genera Leptospermum, Leucopogon, Lissanthe, Pimelea, Pomaderris, and Pultenaea could potentially be used for promoting and sustaining the abundance of bees and beetles in anthropogenic landscapes, such as agriculture and urban areas, in Tasmania and mainland Australia where applicable. However, further empirical tests are required to test the effectiveness of these plants to sustain and promote bee and beetle populations.*

Line 57: What is the significance of months or temporal variation in the context of the proposed research questions? The implications or possible causes of temporal differences and plot-level differences is unclear.

4. Temporal differences were expected to impact the flowering of different plant genera, in turn influencing bee and beetle abundance. Floristic differences across plots could also have a similar effect. These differences could then determine if protected areas support a higher abundance of pollinators than plantations and pastures (question 1).

The influence of plant genera as a spatial and temporal driver of insect pollinator abundance were already discussed in L325-331. The potential influence of plot-level differences was already discussed in L361-367.

Line 160-162; 177: The method used to find ‘best predictive variables’ is unclear. Were two sets of mixed effects models run with different predictors?

5. Correct. We ran two sets of mixed-effect models. The first set was used to determine which predictors had high R-squared values (R^2^) and resulted in model convergence. This was done for each individual predictor. In the second set, these predictors were fitted to a single model. We now explain this approach more in detail:

*L177:* *Instead of using all predictors for model fitting, we only selected those with high R-squared values (R^2^) to find the best predictive variables and ensure model convergence. To do so, we ran two sets of mixed-effect models. Firstly, we fitted a mixed-effect model for each predictor with ‘plot’ set as the random variable, maintaining all other parameters constant. We then calculated the R^2^ values of the model and the fixed and random effects using the rsq R package (68) and finally selected the predictor with non-zero R^2^ values - most predictors had zero R^2^ values indicating that they do not contribute to explaining model variation and were subsequently removed. For categorical predictors (land use and plant genera), we compared the R^2^ between different land use and plant genera and then selected the ones with non-zero values. For continuous predictors (percentage of forest and percentage of open land cover), we compared the R^2^ between different buffers and chose the buffer with the highest R^2^ for both the percentage of forest or open land cover. Finally, after reducing the number of predictors, we fitted a model including all selected predictor variables. We then assessed the predictor estimates and the R^2^ of the model and the fixed and random variables. All statistical analyses were carried out using the R software (64).*
