# Supplementary material for: Association between land use, land cover, plant genera, and pollinator abundance in mixed-use landscapes
Source: PLoS One. 2023 Nov 22;18(11):e0294749. doi: 10.1371/journal.pone.0294749 (PMC10664889; doi:10.1371/journal.pone.0294749)
Supplement: Attachment — Submitted filename: PONE Rebuttal 3.1.docx [file pone.0294749.s003.docx]

**Rebuttal**

Association between land use, land cover, plant genera, and pollinator abundance in mixed-use landscapes

Authors: Vishesh L. Diengdoh, Barry W. Brook, Mark Hunt, Stefania Ondei

Note: The reviewer’s text is in blue while the authors text is in black for clarity. New text by the author is in italics.

**Reviewer #2:**

1. Ln9: It is unclear as to how predictor relevance is an important result

Given that there are numerous predictors, we found that only a few of them were required to explain the variance in the data. This is an important and parsimonious result, because decision makers can make targeted decisions about focusing on monitoring and managing these few predictors to improve pollinator abundance.

We have now reworded the sentence.

Ln9: *Predictors selected (through model selection based on R^2^) and …*

2. Ln10-11: Should it be ‘The land use predictors had a significant effect on honeyeater abundance with protected areas….’

We refrain from using the word “significant” because we have not assessed if a predictor is significant or not using classical null hypothesis significance testing (NHST). Significant effect is generally associated with p-values which we have not calculated in this context. We now use the word “substantive” effect.

L10: *The land-use predictors were selected for only the honeyeater abundance model with protected areas and plantations having substantive positive effects. Land-cover predictors were selected for the honeyeater and native bee abundance models with open land cover within 1500 m and 250 m buffers having substantive negative and positive effects on honeyeaters and native bees respectively.*

3. Ln60: Q3- is related to native vs. invasive comparison; the abstract has no mention of the result obtained for that.

We have now mentioned it in the abstract.

Ln15: plant genera of which only native plants (and not invasive/naturalised) were positively associated with pollinating insects. *Pultenaea* and *Leucopogon* were positively associated …

4. Ln206-217: The uncoupling of graphs and mixed effect model results (tables) makes the results very confusing. I strongly recommend integrating the mixed model results (Ln275-289) with the rest of the results section. Several graphs which have been described in the results section have large variances/standard errors (e.g.: Ln209-210), and it needs to be clarified if those results were statistically significant. The present format of explanation gives the impression that all the described results are significant, which I am afraid is not true.

We respectfully disagree on parts of these combinations, but have done others (see below). Overall, our considered view is that the uncoupling of the figures and tables makes it a lot easier to visualise and interpret the results. Figure 2 and 3 cannot be coupled with any other figure without contrivance. Figures 4-6 have, however, now been combined into a single figure (now Figure 4). Overall, Figures 2-4 show the observational data and it would not be meaningful to couple them with the mixed-effect-modelling results i.e., Tables 1 and 2 and Figure 7. Table 1 and 2 represent different things and it would be confusing to combine the two. However, Table 2 and Figure 7 have now been combined into a single figure (now Figure 5) as they are related and the product of the same model.

We have not made any assessment on statistical significance nor have we stated the results are significant (see response to comment 2). We have simply indicated if a predictor has a positive or negative effect. We do not think significance values would add any value to the results in this study, and the mixing of statistical paradigms like NHST and model selection is frowned upon. The standard errors are adequate to show the precision of the estimates while the R^2^ values give a good idea about the fit of the model.

5. Table S10 provides a summary of the correlation of fixed effects, is the purpose of this correlation to remove effects that were non-independent? or only those with low R2 were excluded?

Correlation of fixed effects is not the same as a correlation between the predictor variables. Rather it is the correlation or covariance between the estimated coefficients. For example, say we take a random sample of the data and carry out the analysis. We will get new estimates of the fixed effects for each random sample. The correlation of fixed effects indicates how many of those fixed effect estimates might be associated. The predictors in Tables S8-11 are those that were used to fit the final model i.e. those with high R^2^., and highly correlated variables will tend to be discarded (this is one important mechanism by which model simplification operates, statistically). It is important to note that the predictors – land use and plant genera are categorial while land cover is numeric.

6. The effect of native vs. invasive plants is done by comparing plant genera (Ln304-306) and not by pooling the values of native plants into one category and then running a statistical analysis on that.

Yes, we compared the effect of the individual plants to show that native plants were positively associated with pollinating insects while invasive/naturalised plants were not. We did not pool the data into native and invasive/naturalised plants and compare the results.
